# The bad and the good of trends in model building and refinement for sparse-data regions: pernicious forms of overfitting versus good new tools and predictions

**DOI:** 10.1107/S2059798323008847

**Published:** 2023-11-03

**Authors:** Jane S. Richardson, Christopher J. Williams, Vincent B. Chen, Michael G. Prisant, David C. Richardson

**Affiliations:** a Department of Biochemistry, Duke University Medical Center, Durham, North Carolina, USA; MRC Laboratory of Molecular Biology, United Kingdom

**Keywords:** overfitting at low resolution, machine-learning predictions, *CaBLAM* validation, *ISOLDE* rebuilding, Ramachandran refinement problems

## Abstract

Model building and refinement, and the validation of their correctness, are very effective and reliable at local resolutions better than about 2.5 Å for both crystallography and cryo-EM. However, at local resolutions worse than 2.5 Å both the procedures and their validation break down and do not ensure reliably correct models. This is because in the broad density at lower resolution, critical features such as protein backbone carbonyl O atoms are not just less accurate but are not seen at all, and so peptide orientations are frequently wrongly fitted by 90–180°. This puts both backbone and side chains into the wrong local energy minimum, and they are then worsened rather than improved by further refinement into a valid but incorrect rotamer or Ramachandran region. On the positive side, new tools are being developed to locate this type of pernicious error in PDB depositions, such as *CaBLAM*, *EMRinger*, Pperp diagnosis of ribose puckers, and peptide flips in *PDB-REDO*, while interactive modeling in *Coot* or *ISOLDE* can help to fix many of them. Another positive trend is that artificial intelligence predictions such as those made by *AlphaFold*2 contribute additional evidence from large multiple sequence alignments, and in high-confidence parts they provide quite good starting models for loops, termini or whole domains with otherwise ambiguous density.

## Abstract 2

The explicit refinement of Ramachandran, rotamer and clash criteria at now-prevalent lower resolutions (2.5–4 Å) has made the current, traditional model validation at the Protein Data Bank nearly useless in this range, since quite poor structures can have perfect scores. Fortunately, new criteria and programs such as *ISOLDE*, *CaBLAM* and *AlphaFold* are coming to the rescue, are already very useful and should be extensible into an effective new community standard.

## 1. Introduction

In structural biology of macromolecules, the lack of atomic detail at low resolution has always been an issue, but it has become a more central problem in recent years because crystallography now routinely solves large, dynamic machines with diffraction limited to 3 Å resolution or lower, while cryo-EM suddenly became capable of solving structures at as high as 3–4 Å resolution (and now even better). This regime is especially problematic because refinement needs more extra information to behave well, and so validation criteria are being used as refinement targets, artificially removing all outliers without trying to correct them. This makes these validation criteria useless, and also turns out to make many of the errors worse rather than better.

‘Low resolution’ or ‘high resolution’ mean different things in different contexts, but for our purposes the distinction happens at resolutions (evaluated locally) where particular critical features change between visible and invisible. For instance in crystallography ‘atomic resolution’ means better than about 1.4 Å, where the saddle points in density between covalently bonded atoms become clear. For nucleic acids, there is a significant transition near 4 Å resolution, where double helices change from continuous density across base pairs with gaps between successive pairs to continuous and much less informative density along the stacking direction. For proteins, an especially critical tipping point for determining backbone conformation is at about 2.5 Å, where the carbonyl O atoms disappear into increasingly featureless tubes of backbone density.

### 1.1. Features visible at different resolutions

**Figure 1:**
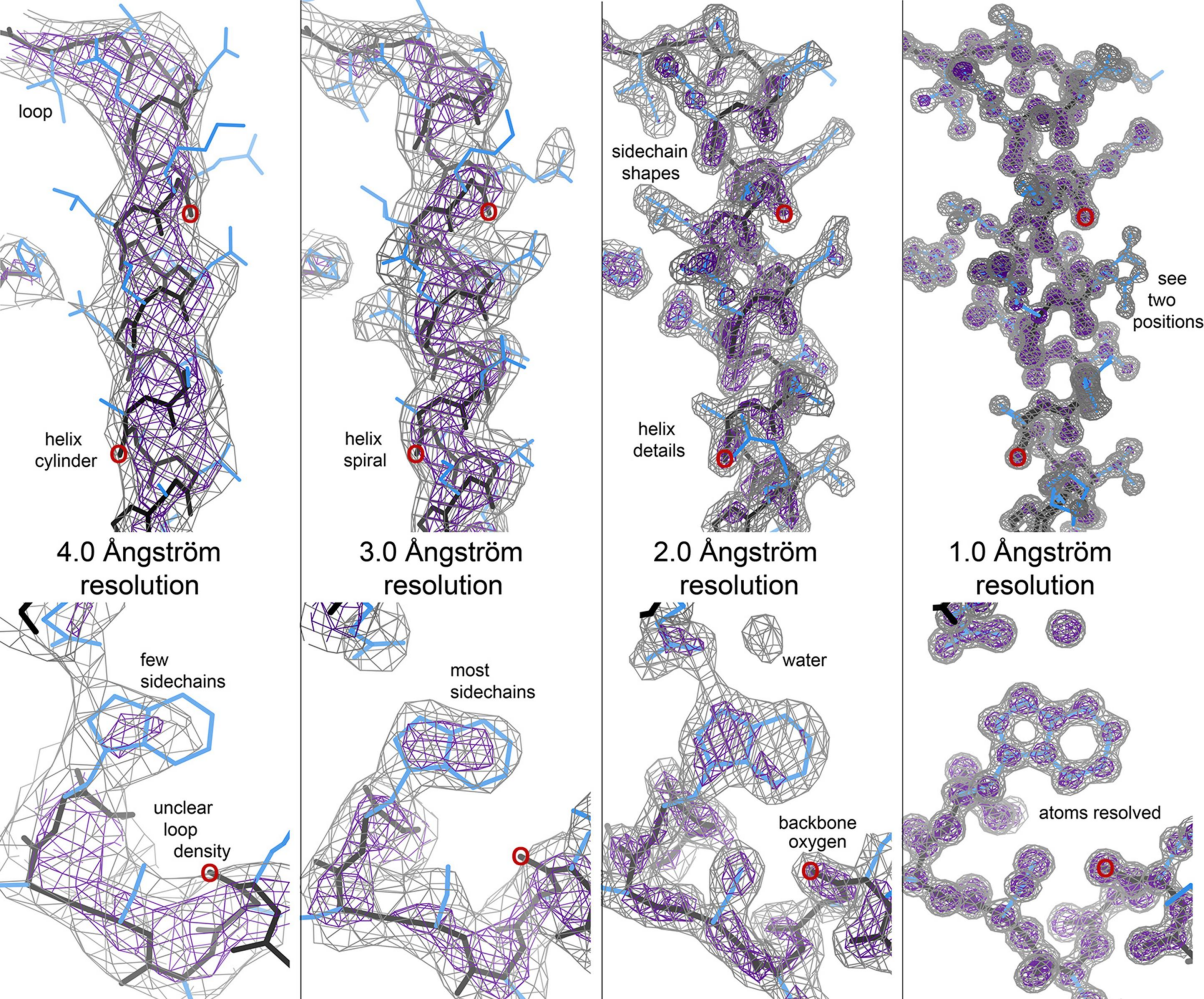
Comparing the same electron-density features at 4, 3, 2 and 1 Å resolution for crystal structures of apo T4 lysozyme. At the top is the helix Ile58–Gly77 and at the bottom is the loop Asn132–Trp138. The left panel is PDB entry 4gbr at 3.99 Å resolution (Zou *et al.*, 2012 ▸), the second panel is PDB entry 5zbh at 3.0 Å resolution (Yang *et al.*, 2018 ▸), the third panel is PDB entry 1lyi at 2.0 Å resolution (Bell *et al.*, 1992 ▸) and the right panel is PDB entry 5jdt at 1.0 Å resolution (Consentius *et al.*, 2016 ▸). All are wild type except for a Thr59→Asp mutation in PDB entry 1lyi. The σ_A_-weighted 2*F*_o_ − *F*_c_ maps are contoured at 1.2σ (gray) and 3σ (purple).

Here, we will systematically visualize electron density as a function of resolution, especially paying attention to the visibility of backbone carbonyl groups and thus of peptide orientation. Fig. 1 ▸ allows a comparison of this effect, and other features, at 4, 3, 2 and 1 Å resolution for a particular helix and a particular loop in apo T4 lysozyme crystal structures, with red ‘O’s marking three specific carbonyl O atoms.

In the well ordered parts of a 1 Å resolution structure, covalently bonded atoms show clearly resolved peaks, allowing bond angles and dihedral angles to be measured accurately, including the orientation of the carbonyls and the peptide planes. Even pairs of alternate conformations are often very clear, as seen for Asn68 on the right-hand side of the helix in the 1 Å panel in Fig. 1 ▸. However, resolution, or relative disorder, is a local property. Ultrahigh-resolution structures almost always have a few disordered stretches at termini or loops, and when model restraints have been greatly loosened or turned off, such parts have the worst geometry outliers in the PDB (Chen *et al.*, 2011 ▸). In great contrast, at 4 Å resolution a helix has become a broad cylinder (see the 4 Å panel in Fig. 1 ▸), the β-sheet is confusing and only a few side chains such as those of tryptophan or selenomethionine can be directly identified. 3–4 Å is actually a difficult resolution range to model well because density connectivity is changing non-uniformly. For instance, a helix goes from a narrow spiral tube with zero density along the helix axis at 2 Å resolution to a wide tube with maximum density along the helix axis at 5 Å resolution. In between, false breaks and false connections are produced by local side-chain mass and hydrogen-bonding details.

In protein crystallography, a structure at 2 Å resolution is considered to be an excellent, workhorse standard. At 2 Å the backbone CO density is a high-contour, clearly directional protrusion out from the main-chain density, as seen for the red-circled O atoms in the 2 Å panel in Fig. 1 ▸. However, at 3 Å resolution there is no CO protrusion even at lower contour levels, the carbonyl O atom is out of density or at its edge, and that density is broadly smooth both along and around the main chain. Thus at 3 Å resolution neither visual inspection nor automated fitting can determine the orientation of the CO group, and thus of the peptide, directly from the experimental data. 2.5 Å is the approximate midpoint of that transition, with some fraction of interpretable nubbins.

By 3 Å resolution side chains have also become problematic, with some atoms outside the shortened, blobby density that no longer shows the characteristic amino-acid shape or its rotamer at all well. Lowering the contour level does not help much with side chains either. Ligand identity and conformation have also become unclear from the density alone, and waters are seldom identifiable.

### 1.2. Current tools

In general, at lower resolutions the fit to density of the model becomes increasingly ambiguous, with multiple distinctly different models giving an equally mediocre fit into the broad and sometimes misleading density, both locally and globally (Richardson, Williams, Videau *et al.*, 2018 ▸). The familiar single-residue, empirical criteria such as Ramachandran, rotamers, ribose puckers *etc.* inherently have multiple minima that pure downhill refinement cannot move between. If multiple models are being compared in model building or rebuilding, then maximizing hydrogen bonding and minimizing some form of atom bumps helps, optimizing all-atom contacts with H atoms is better (Arendall *et al.*, 2005 ▸), and adding Bayesian consideration of prior probabilities would be even better. However, only small local subsets of distinct conformations can feasibly be compared.

A special case is handling rotamers for exposed surface side chains with poor density and no good hydrogen-bond or even van der Waals contacts with anything. Such a side chain has nothing to hold it in an energetically unfavorable conformation (a rotamer outlier). If modeled at all, it should be placed in a favorable rotamer that does not clash with anything and preferably given an occupancy of <1 for the atoms that are poorly seen or unseen.

Recent validation tools aimed at lower resolution problems, such as *CaBLAM* (Williams, Videau *et al.*, 2018 ▸), *EMRinger* (Barad *et al.*, 2015 ▸), Pperp for ribose puckers (Chen *et al.*, 2010 ▸) or *pepflip* in *PDB-REDO* (Joosten *et al.*, 2014 ▸) help by operating on a scale larger than a single residue and/or by evaluating new, less classical parameters that are not yet being explicitly refined. Several new systems specifically tailored for various aspects of cryo-EM include likelihood-based fitting of predictions or fragments into maps (Read *et al.*, 2023 ▸; Millán *et al.*, 2023 ▸), *ModelAngelo* (Jamali *et al.*, 2022 ▸) for maximum-likelihood (ML)-based initial model building and *MEDIC* (Reggiano *et al.*, 2022 ▸) for ML-based validation of final models. These have not yet been broadly tested, but each has been shown to improve on previous methods for its test cases. Other new approaches will hopefully continue to emerge.

High-confidence parts of the new, unprecedentedly successful artificial intelligence (AI) predictions can also help with sparse-data entire structures or regions by providing very good starting hypotheses for the 3D structure, with few geometry or conformational problems. The low-confidence regions are usually bad but are sometimes useful.

## 2. Results and observations

Here, we will draw on our own experience with assessing and correcting problems to provide specific advice for working with lower resolution and locally disordered regions, and will describe useful recent and coming tools and protocols.

### 2.1. Consequences of overfitting, especially peptide orientations

**Figure 2:**
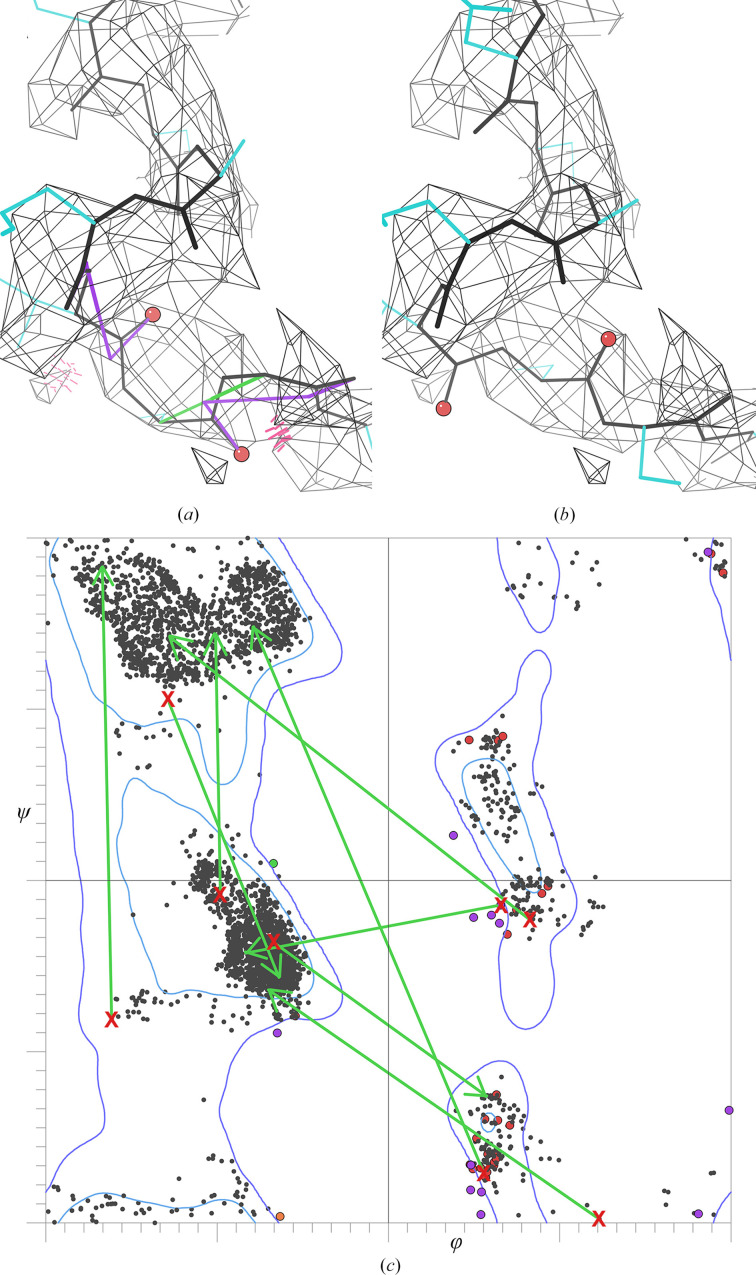
Fixups in PDB entry 6eyc from the misoriented peptides in PDB entry 3ja8. (*a*) Two misoriented CO groups at the end of a helix, flagged by *CaBLAM* outliers and clashes. (*b*) Corrected version, with no outliers, equal map fit and a standard helix C-cap conformation. (*c*) Ramachandran plot for eight residues neighboring misoriented peptides; green arrows point from the misfitted to the corrected positions.

Using the well established Ramachandran, rotamer and other conformational preferences as targets in refinement sounds like a good idea, and some type of conformational restraints are necessary at lower resolution to keep secondary structures and well fitted loops from becoming distorted. However, refining inaccurate initial models against multiple-minimum targets turns out in practice to make the model worse (as shown in Fig. 2 ▸ *c*), in addition to invalidating validation by giving perfect scores for very imperfect structures. As demonstrated and discussed in Section 1, the directionality of peptide CO groups cannot be seen at 3 Å or worse in either X-ray or cryo-EM. As a consequence, the most common error in a backbone trace at such lower resolutions is peptide orientations that are incorrect by large rotations (Prisant *et al.*, 2020 ▸; Lawson *et al.*, 2021 ▸), which means that the φ, ψ values of both surrounding residues will be wrong by as much as 90–180° and subsequently refine into the wrong local minimum in the Ramachandran plot (Croll *et al.*, 2021 ▸). As well as being undefined when the backbone is a smooth tube, peptides are often misoriented at 3–4 Å because of the misleading local breaks or extra connectivity in backbone density described above or by trying to push the carbonyl O atom inside the density contour.

A very useful example structure for the study of such effects is the large MCM2-7 heterohexamer helicase of PDB entry 3ja8 determined at 3.8 Å resolution by cryo-EM (Li *et al.*, 2015 ▸), which had used Ramachandran restraints in refinement and was later carefully studied and corrected (to PDB entry 6eyc) as the test case for the *ISOLDE* user-guided molecular-dynamics (MD) rebuilding program (Croll, 2018 ▸). Those interactive corrections used the AMBER force field, map fit, user judgment and many validation flags, but were independent of the backbone markup in *CaBLAM* (Williams, Videau *et al.*, 2018 ▸). Genuine *cis* nonproline peptides only occur in about one in 3000 residues (Croll, 2015 ▸; Williams, Headd *et al.*, 2018 ▸), and all of the 116 in PDB entry 3ja8 were found to be incorrect, nearly all of them in very poor map density. Especially notably, a sequence misalignment was corrected and 32.5% (1229) of the residues moved a non-H atom by more than 2 Å and/or changed by >45° in φ, ψ or ω.

The first 135 residues in PDB entry 3ja8 (chain 2 residues 201–335) were chosen here as a workable-sized sample for detailed examination (and ended there because residues 340–373 are a weak-density region that was fitted rather differently in PDB entry 6eyc and is unsuitable for close comparisons). Residues 201–335 contain 15 peptide orientations that are wrong by >60°. Two (Leu234 and Thr302) are unjustified *cis* nonprolines. Every one of these 15 is flagged by *CaBLAM*, which validates CO–CO virtual dihedrals in the context of the surrounding five C^α^ atoms. One is a severe C^α^ geometry outlier, eight are at the 1% *CaBLAM* outlier level and six are at the 5% level, while only three are flagged by Ramachandran outliers. In that same region there are three other *CaBLAM* flags at the 5% level. Two of those peptide orientations needed no refitting and the third was rotated in PDB entry 6eyc by a marginal 50°. In this sample, then, all *CaBLAM* outliers were worth looking at, and even at the 5% level (purple markup) two thirds of them were found to be wrong.

Figs. 2 ▸(*a*) and 2 ▸(*b*) show before-and-after CO and peptide orientations for two cases at the end of a helix, both flagged graphically by *CaBLAM* outliers, three-part lines that follow adjacent CO orientations (Prisant *et al.*, 2020 ▸). A misoriented peptide in an initial model changes the preceding ψ angle and the following φ angle by an amount similar to the misfitted peptide rotation angle, thus moving both surrounding φ, ψ positions by very large distances to locations usually within or close to a wrong Ramachandran region. Refinement of the Ramachandran values then pulls them further into the wrong minimum, often removing all Ramachandran outliers but making these model conformations worse rather than better. Fig. 2 ▸(*c*) shows the consequences on a Ramachandran plot for eight residues surrounding badly misoriented peptides, where each green arrow starts at the value in PDB entry 3ja8 and ends at the value in PDB entry 6eyc. All eight of these φ, ψ pairs in PDB entry 3ja8 had been refined into the wrong minimum in Ramachandran space rather than improved. In our anecdotal experience, the residues next to misoriented peptides end up in an incorrect Ramachandran region only somewhat less than 100% of the time.

**Figure 3:**
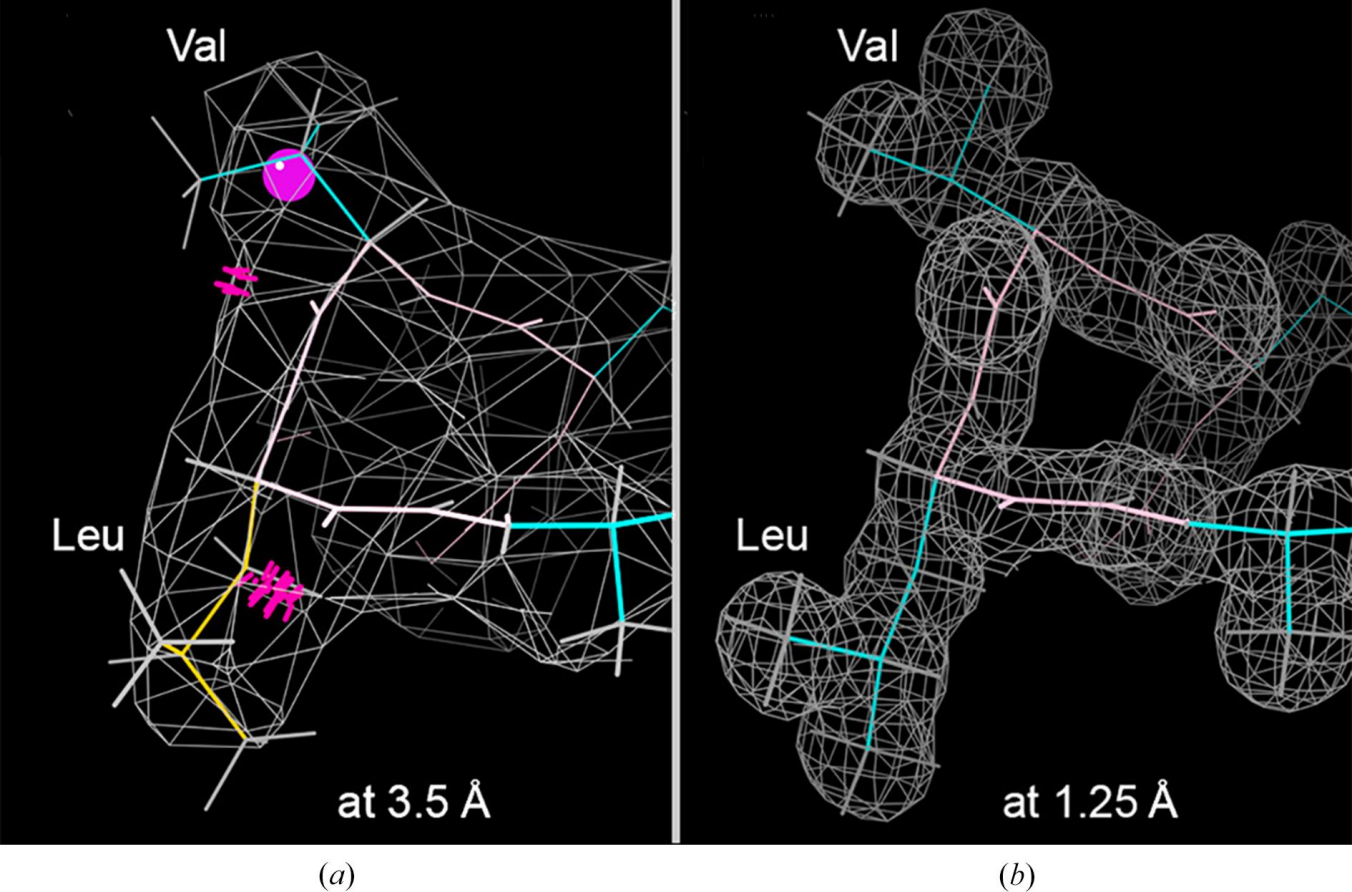
Two side chains pulled too close down into their nubbins of density. (*a*) In PDB entry 2qls at 3.5 Å, with backbone clashes (red spikes), a rotamer outlier (gold) and a C^β^ deviation (magenta ball). (*b*) In PDB entry 2dn2 at 1.25 Å resolution, in clear 2*F*_o_ − *F*_c_ density with relaxed, spread-out rotamers and no outliers.

A similar process happens when incorrect side-chain conformations are refined into the nearest valid rotamer or too slavishly into the map density. One of the most common side-chain errors at low local resolution is that both people and automated systems tend to scrunch side chains down into their nubbins of density, which is seldom the right answer. Fig. 3 ▸ shows two such examples: a helical valine and leucine in hemoglobin structures that are scrunched-down outliers at 3.5 Å and correct in unambiguous density at 1.25 Å resolution.

In addition to the examples above, we have thoroughly documented the many errors in 2.5–4 Å resolution structures and in disordered loops and termini at high resolution by assessments in the EMDB Cryo-EM Challenges (Richardson, Williams, Videau *et al.*, 2018 ▸; Lawson *et al.*, 2021 ▸), comparisons of low-resolution structures with later high-resolution versions (Moriarty *et al.*, 2020 ▸), in our work correcting important structures (Croll *et al.*, 2021 ▸; Dunkle *et al.*, 2011 ▸; PDB entries 6vyo v.2.0, 6m71, 7btf, 7bv1, 7bv2 v.2.0, 5hut v.2.0 and 3q9v v.2.0) and even by local, carefully parallel maximum-likelihood-evaluated refinements between original and corrected versions (Richardson, Williams, Hintze *et al.*, 2018 ▸). This can be a contentious issue because these structures have been made to look misleadingly good by well meaning but dubious procedures that were not obvious to their creators or end users, and no one likes to hear that, including us.

### 2.2. Remedies for overfitting peptide orientations and side chains

When you have an initial model, before refining it consult wider-scale validations including *CaBLAM* and *PDB-REDO* peptide flips for protein and Pperp sugar puckers for RNA, and try to fix the backbone outliers that they show before running refinement. The easiest common *CaBLAM* fixes are (i) idealize an outlier inside secondary structure and (ii) for two *CaBLAM* outliers in a row, try rotating the central CO group. Be aware of the prior improbabilities, such as one in 3000 for a *cis* nonproline (Williams, Videau *et al.*, 2018 ▸) or other very rare conformations. It can also be quite helpful to run all of *PDB-REDO* (Joosten *et al.*, 2014 ▸) or compare with a high-confidence AI prediction to identify candidates for fixing outliers. Accept changes if they are much more probable and/or correct outliers and are about as good a fit to the density. Do not try too hard to get rid of all outliers, and remember that a few outliers are genuine, strained conformations that are conserved by evolution for functional reasons. To prevent distortion in subsequent refinement, it is better to restrain hydrogen bonds rather than Ramachandran scores.

Then check for side chains that are scrunched down into map density as well as those with rotamer outliers, clashes or C^β^ deviation outliers (Lovell *et al.*, 2003 ▸). Try all favorable rotamers to find one that does not clash, preferably makes good contact with something and can have one or two atoms (or even more for long side chains) out of density. Allow the backbone to move slightly when trying different rotamers, either as a backrub rotation around the axis between the *n* − 1 and *n *+ 1 C^α^ atoms (Davis *et al.*, 2006 ▸) or just by letting that large a region adjust in *Coot* (Emsley *et al.*, 2010 ▸) or *ISOLDE*. There are of course other possible problems, but these are the most frequent and most fixable errors. At resolutions poorer than 3 Å, especially for very large structures, the *ISOLDE* user-guided MD rebuilding program with real-time density and validation display (Croll, 2018 ▸), a plug-in to *ChimeraX* (Pettersen *et al.*, 2021 ▸), is the state of the art for correcting problems in either an initial or a post-refinement X-ray, cryo-EM or *AlphaFold* model.

### 2.3. Use of AI predictions at lower resolutions or in poor density

Another new set of useful tools are the highly successful AI structure predictions from *AlphaFold*2 (Jumper *et al.*, 2021 ▸), *RoseTTAFold*2 (Baek *et al.*, 2021 ▸), *RoseTTAFoldNA* (Baek *et al.*, 2022 ▸), *OpenFold* (Ahdritz *et al.*, 2022 ▸), *ESMFold* (Lin *et al.*, 2023 ▸) and others, which bring a rich source of new information from large multiple sequence alignments (MSAs) or large language models. *AlphaFold*2 (AF), the earliest and with the huge, open AlphaFold Database (AFDB) of results (Varadi *et al.*, 2022 ▸) at https://alphafold.ebi.ac.uk/ and the Colab notebook implementation (Mirdita *et al.*, 2022 ▸) at https://bit.ly/alphafoldcolab, is the most used and studied so far and will provide almost all of our examples.

The AF confidence-level measure is called pLDDT (predicted local difference distance test, running from 0 to 100). Models or regions that are predicted with high confidence (pLDDT above 70–75) can very often make better starting models than *de novo* models built into the density at resolutions poorer than 2 Å, for either X-ray or cryo-EM. These should almost never have a stretch of misaligned sequence because estimated residue-pair distances are central to their data, and we have found that when they disagree with alignment in a PDB entry the AF version is always the correct one. They are of course exempt from scrunching side chains down into density since they do not see the density, and they are good at avoiding many other local errors common in experimental starting models. However, they have some new vulnerabilities, such as switching the positions of aliphatic side-chain branches or the positions of long opposed side chains, presumably because these choices are ambiguous in the distances predicted from MSA covariance. Properly pruned pieces of a predicted model (the best pLDDT region) can usually provide molecular-replacement solutions to solve the phase problem for crystal structures (Baek *et al.*, 2021 ▸). All of the major software systems have automated utilities to superimpose a pruned model onto the optimal place in a cryo-EM map and some version of flexible fitting for either technique, as is almost always necessary between domains or for external loops. High-confidence, pruned AF predictions are good starting models for use in *ISOLDE*, and alternatively they can be used as restraints applied to a model from a different data source.

The next step is low-resolution validation and fixup as described in Section 2.2 for experimental starting models, followed by refinement and rebuilding, iterated several times. There is now a new way to improve even further before deposition by cycling back to give prediction implicit information from the experimental data by providing the rebuilt and refined model as a template, which is shown to improve both the predicted models and the models refined from them, converging in about three such cycles (Terwilliger *et al.*, 2022 ▸, 2023 ▸).

**Figure 4:**
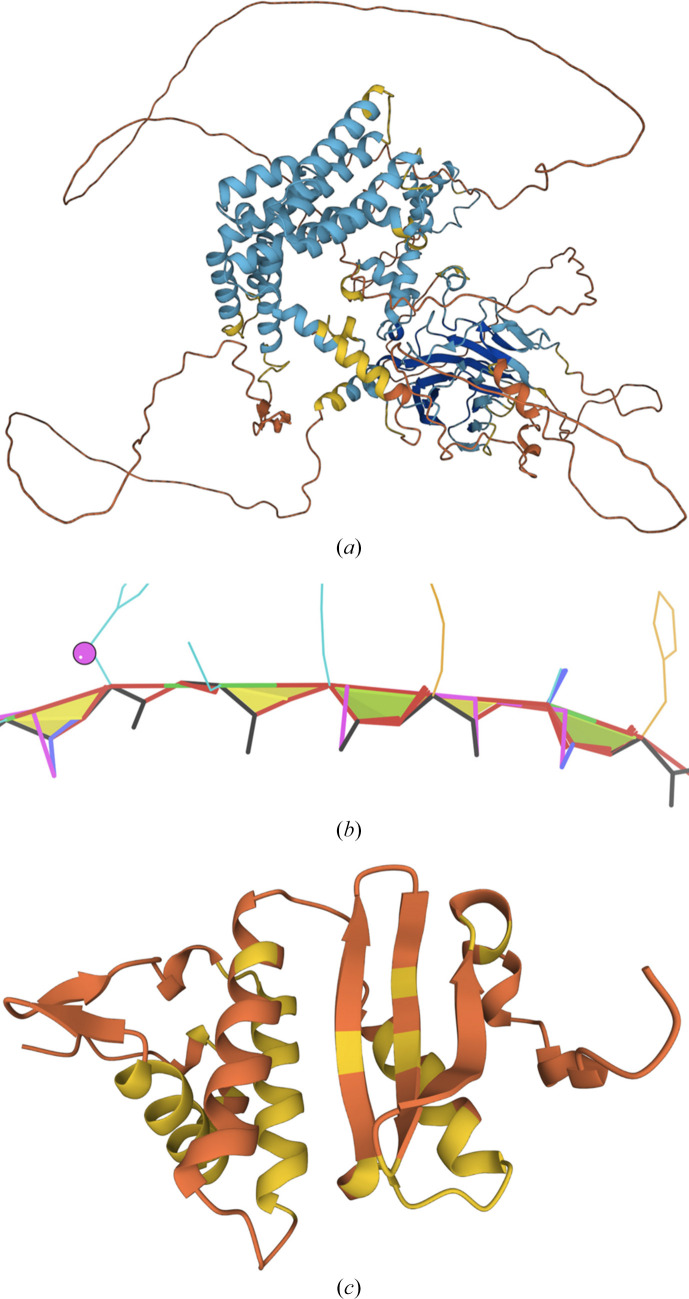
Barbed-wire regions versus near-folded regions in low-pLDDT *AlphaFold* predictions from the EBI AFDB. (*a*) Overview of the AF model for *Saccharomyces cerevisiae* UniProt P53076. (*b*) Close-up of a classic barbed-wire segment in the AF model for human UniProt Q1HG43, with *MolProbity* validation markup. (*c*) A near-folded model for *Methanocaldococcus jannaschii* UniProt Q57775 (pLDDT < 70 in yellow and pLDDT < 50 in orange).

A high-confidence prediction is definitely what one hopes for, but our laboratory is interested in helping less lucky structural biologists to sometimes be able to solve a structure when there is only a mid- or low-confidence prediction. In particular, we have found that even at a local pLDDT under 65 there can be ‘near-folded’ parts that are reasonably compact and protein-like and are known to at least sometimes be good enough for a molecular-replacement solution (Fig. 4 ▸ *c*). However, even more prevalent at pLDDT < 65 are the very different ‘barbed-wire’ parts consisting of long, loopy strands that make essentially no contact with the rest of the model (Fig. 4 ▸ *a*) and with an unprecedented concentration of dire local backbone geometry (Fig. 4 ▸ *b*; Williams *et al.*, 2022 ▸). We hypothesize that barbed-wire regions represent predictions that failed at an early stage, presumably because of unhelpful MSA patterns, and also that barbed wire is the reason that pLDDT < 50 was found to be an excellent predictor of intrinsically disordered regions (IDPs) in CASP14 (Ruff & Pappu, 2021 ▸). As is also true for many IDPs, some barbed-wire regions are known to fold when they find the right binding partner, which can often be shown by *AlphaFold* or *RoseTTAFold* multimer prediction (Drake *et al.*, 2022 ▸). For anyone used to looking at protein 3D structures, barbed-wire segments are obvious in visualizations, especially with outliers turned on, but to enable automation we have developed a set of five specially tuned criteria (packing, ψ, ω, *CaBLAM* and geometry) that can identify and delete the barbed wire from low-pLDDT regions, leaving the near-folded parts that may have usable predictive value, as in Fig. 4 ▸(*c*). A preliminary version of this tool is available on the *Phenix* command line as barbed_wire_analysis and will be further tested and improved.

## 3. Conclusions

At resolutions significantly worse than 2.5 Å, traditional model validations of Ramachandran, rotamer and all-atom clash outliers become nearly useless because the outliers can be artificially refined away within the broad density without correcting the underlying problems and without compromising the *R* factors or other fit-to-data and fit-to-density measures. Both the structural biology community and the PDB need to address this issue as promptly as possible. We should use the new tools that are now available to identify and correct bad local conformations before refinement sweeps them under the rug, and the PDB ‘slider’ summaries and detailed validation reports need to include new model and fit-to-map validation metrics, as recommended by the Cryo-EM Task Force and community meeting at Hinxton in January 2020.

Individual new structure determinations can take advantage of starting models from AI predictions and of validations such as *CaBLAM*, *EMRinger*, Pperp ribose pucker and peptide-flip diagnosis, and then use correction tools such as *Coot*, *PDB-REDO* and/or *ISOLDE* both before and after refinement and rebuilding.
